# Maize Cultivation and Improvement

**DOI:** 10.3390/plants15050794

**Published:** 2026-03-04

**Authors:** Glauco Vieira Miranda

**Affiliations:** Department of Agronomy, Federal University of Technology Paraná, Santa Helena 85892-114, Brazil; glaucovmiranda@utfpr.edu.br

This Special Issue “Maize Cultivation and Improvement” gathers eight high-quality contributions that collectively reflect the contemporary landscape of maize research. These articles span molecular genetics, quantitative breeding, stress physiology, proteomics, genomic prediction, agronomic management, artificial intelligence, and meta-analytical synthesis. Together, these articles illustrate how modern maize improvement is increasingly interdisciplinary, integrating biotechnology, data science, and field-based validation to accelerate genetic gain and enhance sustainability.

Maize (*Zea mays* L.) remains one of the most strategically important crops worldwide, serving as food, feed, fiber, and fuel. However, climate variability, increasing biotic and abiotic stresses, and the demand for higher productivity under resource constraints require transformative approaches. The studies compiled in this volume demonstrate how germplasm development, precision phenotyping, and advanced analytics can converge to address these global challenges.

## 1. Molecular Regulation and Functional Genetics

Understanding the genetic and molecular mechanisms underlying key agronomic traits remains foundational to crop improvement. Two studies in this Special Issue provide important advances in this direction.

The identification of a single-base mutation in the *Dwarf 1* (D1) gene responsible for reduced plant height offers valuable insight into plant architecture control [1]. Plant height directly influences lodging resistance, biomass allocation, and yield stability. The identification of this mutation provides a potential target for marker-assisted selection and functional validation strategies aimed at optimizing canopy structure in diverse environments.

Similarly, the investigation of cysteine protease ZmPCP in regulating pollen viability, pollen tube growth, and seed development addresses reproductive resilience under drought and heat stress [2]. Reproductive success is one of the most climate-sensitive phases in maize production. By linking molecular regulation to pollination efficiency and seed formation, the study contributes to the development of germplasm that is better adapted to extreme environmental conditions.

## 2. Stress Physiology and Proteomic Responses

Climate change intensifies the frequency of combined drought and heat stress events. The comparative leaf proteome analysis of maize under simultaneous drought and heat stress provides a system-level understanding of stress tolerance mechanisms [3]. The screening of 45 inbred lines and identifying contrasting proteomic responses highlights the importance of integrating physiological evaluation with omics technologies. Such approaches enable the identification of candidate pathways and biomarkers for stress resilience.

The work reinforces the necessity of coupling molecular-level insights with breeding strategies, allowing selection decisions to move beyond purely phenotypic evaluation toward mechanistic understanding.

## 3. Quantitative Breeding and Genetic Gain

Sustained genetic progress depends on efficient selection methodologies. A study on genetic gains with a field validation of synthetic tropical maize populations demonstrates the power of selection indexes combined with REML/BLUP methodology [4]. By evaluating recurrent selection strategies under tropical conditions, the study underscores the importance of robust statistical modeling in managing genotype–phenotype interactions and inbreeding effects.

Complementing this, a work on enhancing across-population genomic prediction for maize hybrids addressed a central limitation of genomic selection: the prediction accuracy across heterotic groups and breeding populations [5]. Improving cross-population prediction expands the operational scope of genomic selection and reduces breeding cycle length, directly contributing to accelerated genetic gain.

Together, these studies illustrate the evolution from classical recurrent selection to data-driven genomic breeding pipelines, reinforcing the integration of statistical genetics and computational methodologies in modern maize improvement programs.

## 4. Agronomic Management and Environmental Interactions

Genetic potential can only be realized through optimized management. A study evaluating the effects of semi-arid environmental conditions and agronomic traits on grain quality emphasizes the interaction between genotype and environment in determining both yield and grain composition [6]. As production expands into marginal or stress-prone regions, understanding how environmental variables influence quality parameters will become increasingly important for food and feed industries.

At a broader scale, the meta-analysis on increased planting density in Northeast China synthesizes evidence from 508 paired observations [7]. By quantitatively assessing yield responses to planting density adjustments, the work provides a statistically robust framework for optimizing management strategies. The integration of large-scale evidence through a meta-analysis strengthens agronomic recommendations and supports region-specific decision-making.

## 5. Digital Agriculture and High-Throughput Phenotyping

One of the most forward-looking contributions in this Special Issue is a CEHD framework for detection and height estimation of fresh corn ears under field conditions [8]. By applying deep learning architectures (YOLO-based models) for real-time ear detection and height estimation, the research exemplifies the integration of artificial intelligence into crop production systems.

Precision detection supports dynamic harvester adjustment, reduces mechanical damage, and improves harvest efficiency. More broadly, the study signals the transition toward Industry 4.0 in maize production, where visual computing, machine learning, and real-time analytics enhance operational precision and reduce post-harvest losses.

## 6. Convergence of Technologies in Maize Improvement

Across all eight papers, a clear theme emerges: the convergence of biotechnology, quantitative genetics, omics platforms, high-throughput phenotyping, and artificial intelligence.

The key integrative advances highlighted in this volume include:

- Functional gene identification linked to agronomic performance.
- The proteomic and molecular characterization of stress tolerance.
- REML/BLUP and selection indices to enhance recurrent selection efficiency.
- Genomic prediction models extending across breeding populations.
- Evidence-based agronomic optimization via meta-analysis.
- AI-driven field phenotyping for harvest precision.

This convergence allows shortened breeding cycles, reduced experimental population size requirements, enhanced prediction accuracy, and strengthened translation of genetic knowledge into field performance.

## 7. Future Directions

Looking ahead, maize improvement will increasingly rely on the following:

1. Multi-omics integration (genomics, transcriptomics, proteomics, phenomics).
2. Climate-resilient germplasm development targeting combined stress scenarios.
3. Scalable genomic prediction models across heterotic groups.
4. Digital agriculture platforms enabling real-time crop monitoring.
5. Data-driven agronomic optimization supported by large-scale synthesis approaches.

The integration of these domains will be critical for achieving sustainable yield increases while maintaining environmental stewardship and economic viability.

## 8. Concluding Remarks

The contributions compiled in this Special Issue reflect the dynamism in maize research and the collaborative efforts of scientists worldwide. By combining molecular biology, advanced statistics, computational intelligence, and field validation, these studies collectively advance the theoretical and applied dimensions of maize cultivation and improvement.

I would like to sincerely thank all authors, reviewers, and the editorial team for their dedication and scientific rigor. The success of this Special Issue demonstrates the vitality of interdisciplinary research in maize and sets the foundation for continued innovation in germplasm development, breeding strategies, and sustainable production systems.

The publication of this Special Issue in book format provides a consolidated reference for researchers, breeders, agronomists, and students engaged in advancing maize science. I hope that the insights presented here will contribute meaningfully to global food security and to the continued evolution of maize breeding in an era defined by climatic uncertainty and technological transformation.
